# Acute Bilateral Ophthalmoparesis with Pupilary Areflexical Mydriasis in Miller-Fisher Syndrome Treated with Intravenous Immunoglobulin

**DOI:** 10.1155/2010/291840

**Published:** 2010-09-01

**Authors:** Theocharis Papanikolaou, Cath Gray, Bernard Boothman, Gerald Naylor, George Mariatos

**Affiliations:** ^1^Ophthalmology Department, Addenbrooke's Teaching Hospital, Hills Road, Cambridge CB2 0QQ, UK; ^2^Ophthalmology Department, Blackpool Fylde and Wyre Hospitals, Whinney Heys Road, Blackpool FY3 8NR, UK; ^3^Neurology Department, Blackpool Fylde and Wyre Hospitals, Whinney Heys Road, Blackpool FY3 8NR, UK; ^4^Ophthalmology Department, Barnsley General Hospital, Gawber Road, Barnsley S75 2EP, UK

## Abstract

Miller-Fisher syndrome (MFS) is a rare condition characterized by the classical triad of ophthalmoplegia, ataxia, and areflexia (Fisher, 1956). It is considered a variant of Guillain-Barré syndrome (GBS) with which it may overlap, or it can occur in more limited forms. We report a case of a thirty-five-year-old male who presented with a six-day history of diplopia, following a recent chest infection. On examination, he was found to have bilateral sixth nerve palsy, bilateral fourth nerve palsy, bilateral areflexical mydriasis, ataxia and total absence of reflexes. After excluding other conditions, a diagnosis of Miller-Fisher syndrome was made. The patient was administered intravenous immunoglobulin and made an uneventful recovery.

## 1. Case Report

A thirty-five-year-old male patient presented to our department complaining of diplopia of 6 days duration. He was generally fit and well with no significant past medical history. Ten days previously, he had suffered a chest infection for which he had a course of antibiotics and had made a good recovery.

On examination, his visual acuity was 6/5 unaided bilaterally.

Colour vision tested with Ishihara plates was normal, and visual field testing by confrontation was also normal.

**Figure 1:**
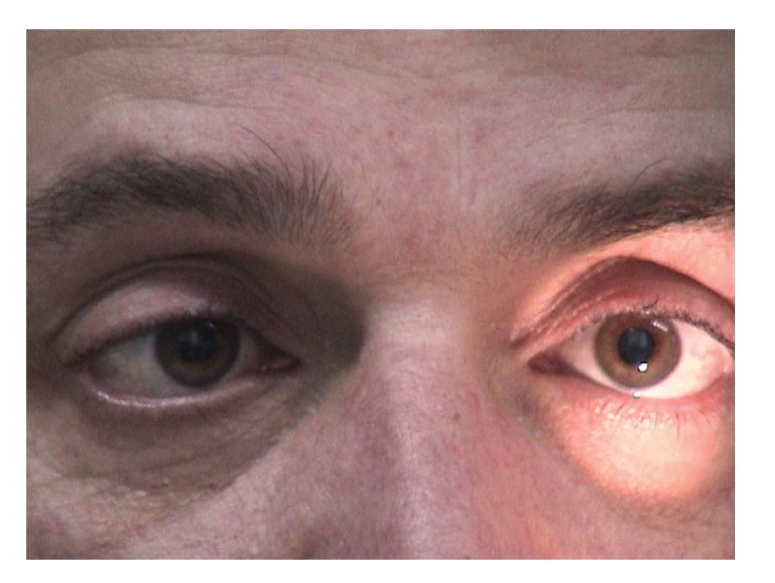
Bilaterally dilated pupils with absent response to light when light is shown to the left eye.

**Figure 2:**
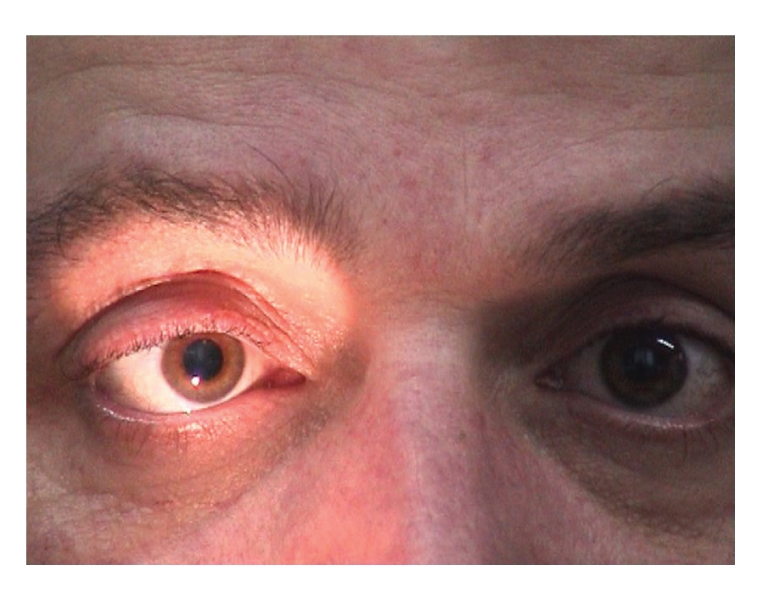
Bilaterally dilated pupils with absent response to light when light is shown to the right eye.

**Figure 3:**
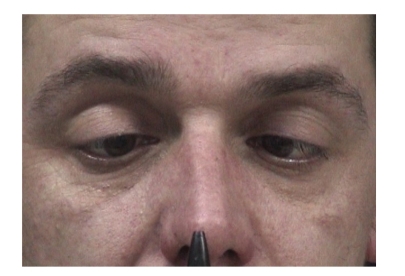
Absent pupil response to accommodation of both eyes and normal convergence.

Both pupils were dilated and were not reacting to light and accommodation. (Figures 1, 2, and 3).

Ocular motility testing revealed signs suggesting bilateral fourth nerve palsy and bilateral sixth nerve palsy.

The remainder of the cranial nerves were intact.

Anterior segment examination and fundoscopy were normal on both sides.

**Figure 4:**
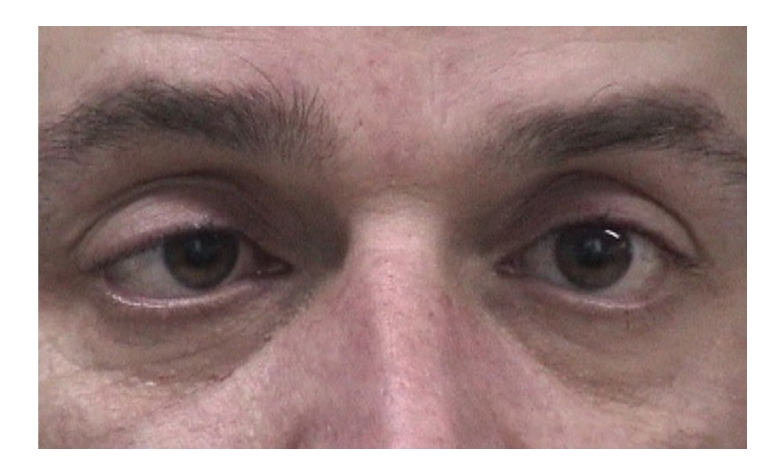
Right esotropia.

**Figure 5:**
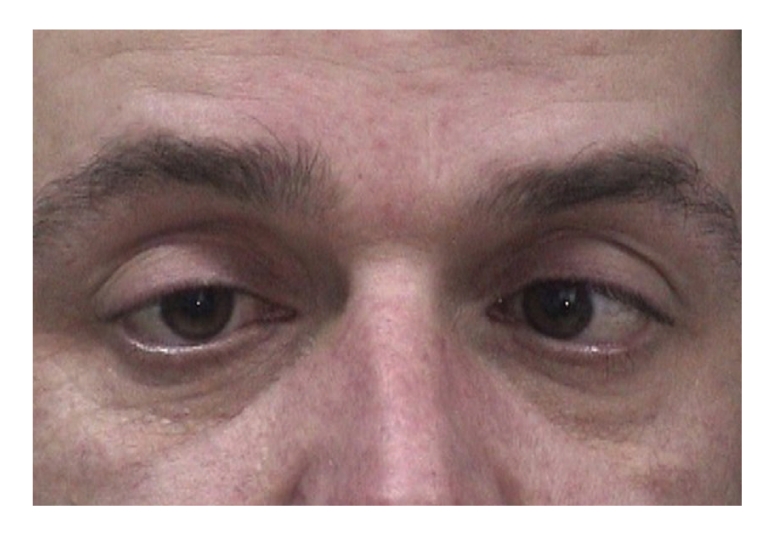
Left esotropia.

**Figure 6:**
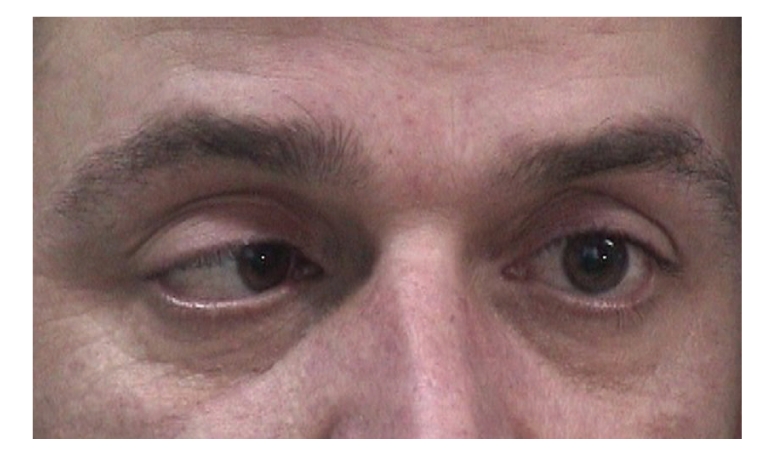
Underaction of left eye on laevoversion.

**Figure 7:**
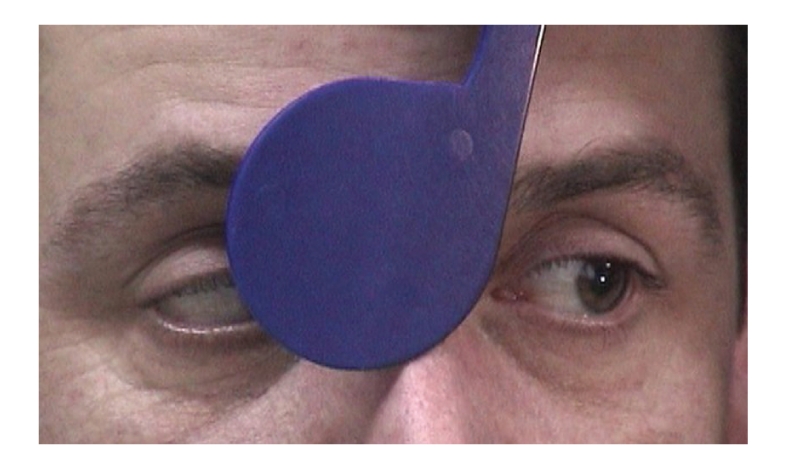
Full abduction of left eye on ductions.

**Figure 8:**
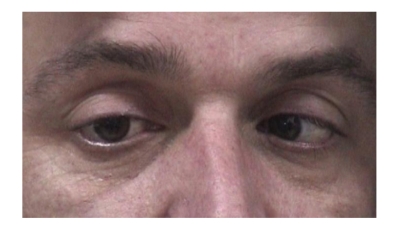
Underaction of right eye on dextroversion.

**Figure 9:**
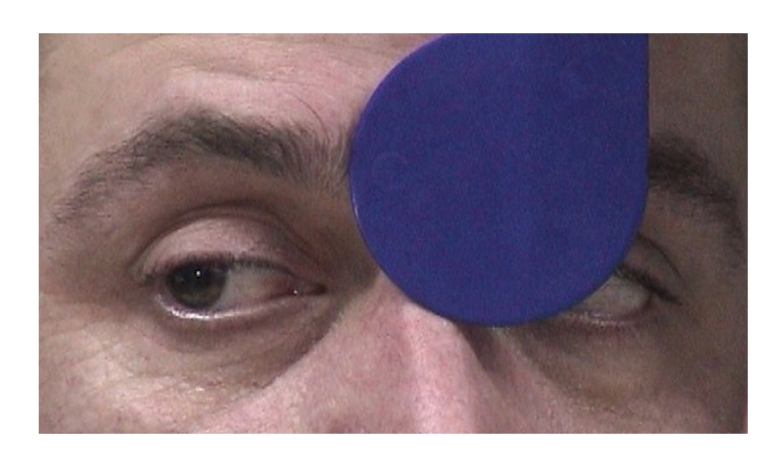
Full abduction of right eye on ductions.

**Figure 10:**
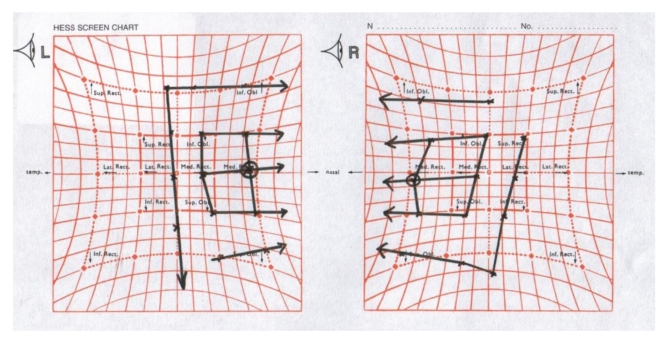
Hess chart showing bilateral fourth and sixth nerve palsy.

All his reflexes were absent and he had truncal unsteadiness when carrying out heel-to-toe walking. A formal orthoptic assessment was carried out which verified the presence of bilateral fourth and sixth nerve palsy (Figures 4, 5, 6, 7, 8, 9, and 10).

Pharmacological testing with 0,125% Pilocarpine caused miosis on both sides.

Subsequently, the patient had a brain MRI scan that did not reveal any abnormalities.

On the basis of the clinical findings a diagnosis of Miller Fisher syndrome was made. Serum samples were tested for anti-GQ1b ganglioside antibodies and an elevated IgG titer was reported.

The patient was referred to a neurologist. He did not undergo CSF examination. He was administered intravenous immunoglobulin and made an uneventful recovery within ten weeks.

## 2. Discussion

Miller-Fisher syndrome is considered a variant of Guillain-Barré syndrome.

It was first described in 1956 as a triad of ophthalmoplegia, ataxia, and areflexia [1].

The pathogenesis is considered immunological [2], molecular mimicry being the most likely mechanism [3].

Anti-GQ1b ganglioside antibodies have been shown to be a serum marker for MFS [4]. They can be found in 85% of cases and have been proposed as one of the diagnostic criteria for MFS. An MRI of the brain is not necessary to make the diagnosis which can be made solely on clinical grounds having excluded other pathology [5]. Usually, abnormal high intensity lesions of the brainstem, thalamus, cerebellum, and cerebrum are seen on MRI in patients with Bickerstaff's brainstem encephalitis with ophthalmoplegia, ataxia and hypereflexia [6].

The differential diagnosis also includes botulism [7], cerebrovascular accident, myasthenia gravis, and Wernicke's encephalopathy with the latter being the one closer mimicking MFS, usually presenting with mental changes, ataxia, and extraocular muscle paralysis with nystagmus in a person with nutritional deficiency precipitated by infection, gastrointestinal disorder or trauma [8].

A variety of infections can precede the onset of symptoms [9].

The full triad of MFS is not always present [5] and there might be an overlap with GBS [10]. Mydriasis which exhibits denervation hypersensitivity with or without light-near dissociation is also not always present [11].

The natural recovery of the condition is very good in the majority of cases with or without treatment [12].

There are no randomised controlled trials of immunomodulatory therapy in MFS on which to base practice yet [13]. The role of plasmapheresis in the treatment of MFS is controversial. Some studies have shown benefit [14] while others failed to do so [15]. Intravenous immunoglobulin has been shown to slightly hasten the periods between ophthalmoplegia and ataxia onset and the start of the alleviation of these symptoms [16].

Ophthalmologists should maintain a high level of suspicion of MFS as they may be the first doctors to encounter this condition.

Careful history taking and the bilaterality of the condition are keys to the diagnosis even though unilateral cases have been reported. After other pathology has been excluded, a prompt referral to a neurologist is mandatory as the condition may progress to GBS [12] with devastating consequences.
